# (Ca_0_._25_La_0_._5_Dy_0_._25_)CrO_3_ Ceramic Fiber@Biomass-Derived Carbon Aerogel with Enhanced Solute Transport Channels for Highly Efficient Solar Interface Evaporation

**DOI:** 10.3390/ma17102205

**Published:** 2024-05-08

**Authors:** Wei Zhang, Liyan Xue, Jincheng Zhang, Meng Zhang, Kaixian Wang, Minzhong Huang, Fan Yang, Zhengming Jiang, Tongxiang Liang

**Affiliations:** 1 School of Materials Science and Engineering, Jiangxi University of Science & Technology, Ganzhou 341000, China; xmzhangwei@fjirsm.ac.cn; 2 Fujian Institute of Research on the Structure of Matter, Chinese Academy of Sciences, Fuzhou 350002, China; xmxueliyan@fjirsm.ac.cn (L.X.); xmzhangjincheng@fjirsm.ac.cn (J.Z.); zmjn18@163.com (M.Z.); xmwangkaixian@fjirsm.ac.cn (K.W.); 3 Xiamen Institute of Rare Earth Materials, Haixi Institutes, Chinese Academy of Sciences, Xiamen 361021, China; 4 Fujian Science & Technology Innovation Laboratory for Optoelectronic Information of China, Fuzhou 350108, China; 5 Xiamen Key Laboratory of Rare Earth Photoelectric Functional Materials, Xiamen Institute of Rare Earth Materials, Haixi Institutes, Chinese Academy of Sciences, Xiamen 361021, China; 6 Key Laboratory of Rare Earths, Ganjiang Innovation Academy, Chinese Academy of Sciences, Ganzhou 341000, China; 7 China Nuclear Power (Shanghai) Simulation Technology Co., Ltd., Shanghai 200241, China; 8 College of Rare Earths, Jiangxi University of Science & Technology, Ganzhou 341000, China

**Keywords:** rare earth, ceramic fiber, aerogel, solar evaporation, salt resistance

## Abstract

The use of solar interface evaporation for seawater desalination or sewage treatment is an environmentally friendly and sustainable approach; however, achieving efficient solar energy utilization and ensuring the long-term stability of the evaporation devices are two major challenges for practical application. To address these issues, we developed a novel ceramic fiber@bioderived carbon composite aerogel with a continuous through-hole structure via electrospinning and freeze-casting methods. Specifically, an aerogel was prepared by incorporating perovskite oxide (Ca_0_._25_La_0_._5_Dy_0_._25_)CrO_3_ ceramic fibers (CCFs) and amylopectin-derived carbon (ADC). The CCFs exhibited remarkable photothermal conversion efficiencies, and the ADC served as a connecting agent and imparted hydrophilicity to the aerogel due to its abundant oxygen-containing functional groups. After optimizing the composition and microstructure, the (Ca_0_._25_La_0_._5_Dy_0_._25_)CrO_3_ ceramic fiber@biomass-derived carbon aerogel demonstrated remarkable properties, including efficient light absorption and rapid transport of water and solutes. Under 1 kW m^−2^ light intensity irradiation, this novel material exhibited a high temperature (48.3 °C), high evaporation rate (1.68 kg m^−2^ h^−1^), and impressive solar vapor conversion efficiency (91.6%). Moreover, it exhibited long-term stability in water evaporation even with highly concentrated salt solutions (25 wt%). Therefore, the (Ca_0_._25_La_0_._5_Dy_0_._25_)CrO_3_ ceramic fiber@biomass-derived carbon aerogel holds great promise for various applications of solar interface evaporation.

## 1. Introduction

Water resources are indispensable for human survival and social development. The majority of Earth’s water resources consist of seawater, which is challenging to utilize directly, while only 2.5% of the available freshwater can be used directly. This significant disparity between the supply and demand of freshwater poses a serious challenge to human existence, necessitating resolution of the freshwater scarcity issue [1,2,3,4]. Conventional methods for freshwater production primarily involve reverse osmosis (RO) and low-temperature multieffect distillation (MED), both of which consume substantial energy and emit pollutants during production processes, thereby impeding sustainable production of clean water [5,6]. In recent years, solar interface evaporation technology has become a viable solution for seawater desalination and wastewater purification by utilizing sunlight as the sole energy source. By harnessing the photothermal effect to concentrate heat at the air–liquid interface, this technology facilitates efficient water evaporation while addressing concerns related to clean water production and energy consumption [7].

Solar interface evaporation is facilitated by solar evaporator devices, which must satisfy two prerequisites [8,9]: (i) Efficient absorption over a wide range of solar radiation and (ii) conversion of light energy into heat energy to induce liquid evaporation. Based on the principles governing light-to-heat transfer in evaporator materials, they can be broadly categorized as plasmonic metals, carbon-based materials, and semiconductors. Plasmonic metals increase the surface temperatures of materials by harnessing light energy through localized surface plasmon resonance [10], with typical examples including Au NPs [11], Ag NPs [12], AgNi [13], etc. However, metal materials exhibit high absorption only at specific wavelengths, leading to relatively low overall photothermal efficiencies [14]. Moreover, the practical application of precious metals is constrained by their costs. Carbon materials play crucial roles in generating molecular thermal vibrations [15] and have garnered significant attention due to their abundance, cost-effectiveness, and remarkable efficacies. For instance, Xu et al. [16] devised an evaporator comprising carbon nanotubes (CNTs)@carbon black (CB)/polyvinyl alcohol (PVA), which exhibited exceptional resistance to salt crystallization. After 12 h of irradiation at 2.0 kW m^−2^, the evaporation rate stabilized at 2.58 ± 0.10 kg m^−2^ h^−1^. Li et al. [17] obtained biomass-derived carbon aerogels by carbonizing nanocellulose and showcased its three-dimensional network structure and low thermal conductivity, which provided light absorption efficiencies of 94–97% and an evaporation rate of 1.54 kg m^−2^ h^−1^ under 1 kW m^−2^ radiation. Wu et al. [18] synthesized a porous network aerogel by crosslinking plantain cellulose (PC) with biomass hollow carbon tubes (HCTs), which provided excellent salt tolerance and high evaporation rates under solar illumination at 1.0 kW m^−2^. Notably, semiconductor materials, owing to their unique optical properties, convert light energy into heat energy through nonradiative relaxation of electron–hole pairs returning to the ground state after photoexcitation, which has garnered significant attention from researchers [19,20,21,22,23]. Yang et al. [21] achieved enhanced solar energy absorption with λ-Ti_3_O_5_ (96.4%) by adjusting the combined state density and subsequently combining it with a porous hydrogel to fabricate an evaporator, which exhibited evaporation rates of up to 6.09 kg m^−2^ h^−1^ in simulated seawater under at a 1 kW m^−2^ light intensity. Zhang et al. [22] constructed a three-dimensional evaporator by combining Zn-doped MoS_2_ with modified sorghum straw, demonstrating a high evaporation rate of 3.46 kg m^−2^ h^−1^ under 1 kW m^−2^ irradiation. Zhao et al. [23] prepared a CuS-modified attapulgite composite aerogel with potato starch biochar as a binder, which exhibited excellent photothermal effects and achieved an evaporation rate of 1.57 kg m^−2^ h^−1^ under 1 kW m^−2^ radiation while also displaying remarkable salt tolerance. Liu et al. [20] synthesized a Ti_3_C_2_T_x_/carbon aerogel with inexpensive wax gourd as the biocarbon precursor material; due to a synergistic effect from the MXenes and carbon aerogel components, the Ti_3_C_2_T_x_/carbon aerogel exhibited satisfactory photothermal conversion efficiency (92.3%) and cyclic stability while achieving an evaporation rate of 1.48 kg m^−2^ h^−1^ with one sun illumination.

Electrospinning is a simple and efficient method to prepare fiber-based aerogels with regular fiber morphology, large specific surface area, and complex pore structure, so it has obtained applications in many fields. For example, Wang et al. [24] used electrospinning and carbonization processes to produce CO_2_ adsorbed carbon fiber aerogels with excellent adsorption capacity and cycle stability. Karan et al. [25] embedded the copper cystine hybrid into the electrospun nanofiber aerogels, which is expected to completely change tissue regeneration and wound healing. Hasan et al. [26] used electrospinning polyacrylonitrile (PAN) and biochar to construct mesoporous microporous carbon electrode aerogel. The aerogel electrode material has excellent cycle stability and a specific capacitance of 407 F/g, which has great application potential in high-performance supercapacitors in the future. Fiber-based aerogels also have good thermal insulation effects and mechanical properties. Karamikamkar et al. [27] used electrospinning thermoplastic polyurethane (TPU) nanofibers as the aerogel framework and polymerized silica precursor to produce composite aerogels with excellent mechanical properties and thermal insulation effects. Nanofiber aerogels can realize functional expansion after being compounded with other materials. Rahmanian et al. [28] innovatively synthesized metal organic framework materials (MOF) on electrospun porous nanofiber aerogel (NFA) so that composite materials have functional diversity. Liu et al. [29] developed polyaniline (PANI) modified polyarylene ether nitrile (PEN) electrospinned nanofiber membranes (PANI@PEN) that can be used in the field of seawater desalination with an evaporation rate of 1.527 kg m^−2^ h^−1^.

La_1−x_Ca_x_CrO_3_ ceramics, representative infrared-responsive semiconductor materials, have been extensively investigated and applied in optics [30,31,32] due to their wide spectral absorption ranges, excellent corrosion resistances, high stability, and moderate costs. Remarkably, Lu et al. [30] reported that the La_0_._5_Ca_0_._5_CrO_3_ powder they synthesized exhibited exceptional light absorption efficiencies of up to 95% within the wavelength range of 0.2–3 μm. Moreover, with a light intensity of 1 W cm^−2^, the surface temperature rapidly increased from room temperature to 190 °C within just 25 s, demonstrating an extremely rapid photothermal response and efficient photothermal conversion capability. Therefore, it is suggested that La_1−x_Ca_x_CrO_3_ ceramics hold great potential for use in interface evaporation. In the present study, (Ca_x_La_y_Dy_1−x−y_)CrO_3_ ceramic fibers were initially prepared with electrospinning technology, and ceramic fiber@biomass-derived carbon composite aerogels were prepared with continuous through-hole structures via freeze-casting and amylopectin as a green binder. Furthermore, the microstructures and optical properties of the (Ca_x_La_y_Dy_1−x−y_)CrO_3_ ceramic fibers were investigated, the ratios of ceramic fibers to amylopectin were determined, and the evaporation effects, stabilities, and desalination of the composite aerogels were examined in simulated seawater. This study presents an innovative material for use in seawater desalination and sewage treatment while expanding the range of material selection.

## 2. Materials and Methods

### 2.1. Materials

Lanthanum nitrate, chromium nitrate, dysprosium nitrate, calcium nitrate, yttrium nitrate, cesium nitrate, praseodymium nitrate, and erbium nitrate, as well as polyvinylpyrrolidone (PVP) and anhydrous ethanol, were purchased from Aladdin Industrial Co., Ltd. (Shanghai, China); N,N-dimethylformamide (DMF) was sourced from Aladdin Industrial Co., Ltd., Shanghai, China; amylopectin (AP) and ammonium persulfate were also obtained from Aladdin Industrial Co., Ltd., Shanghai, China; and N,N-methylenebisacrylamide was obtained from Macklin Industrial Co., Ltd. (Shanghai, China). The above drugs are all analytically pure.

### 2.2. Synthesis of (Ca_0.25_La_0.5_Dy_0.25_)CrO_3_ Ceramic Fibers

Firstly, the nitrate of the four components was dissolved in a certain proportion of anhydrous ethanol, ultrapure water, and DMF according to the stoichiometric ratio. In addition, 8 wt% PVP was added to the fully mixed solution and stirred for 12 h to form a spinning solution with considerable viscosity. The experiment was conducted on electrospinning equipment (NANON-1A, MECC, Fukuoka, Japan) under the condition of 19 kV, with the needle 15 cm away from the collection plate, and the precursor solution was injected at a speed of 0.6 mL h^−1^. The external environment was controlled at 30 °C and 25% humidity. The spun raw silk fiber was first dried in air at 80 °C for 1 h, then heated in a muffle furnace at 1 °C min^−1^ from room temperature to 500 °C and 8 °C min^−1^ from 500 °C to 1000 °C, and then heated at 1000 °C for 15 min to obtain (Ca_0_._25_La_0_._5_Dy_0_._25_)CrO_3_ ceramic fiber samples [33,34]. (Ca_0_._2_La_0_._6_Dy_0_._2_)CrO_3_, (Ca_0_._33_La_0_._33_Dy_0_._33_)CrO_3_ and Ca_0_._2_La_0_._8_CrO_3_ were prepared by the same method as the control group for the light absorption effect.

### 2.3. Preparation of Ceramic Fiber@Biomass-Derived Carbon Composite Aerogel

After determining the composition of the evaporator light absorbent, we selected amylopectin (AP) as the auxiliary molding agent of the ceramic fiber. Amylopectin is widely found in various biomass starches and is a widely available, inexpensive, and highly abundant biomass material. It has excellent adhesion and forming effects and is an ideal source material for biomass-derived carbon aerogel [35,36,37]. The specific preparation steps [38,39]: First, the ceramic fibers were crosslinked by high-speed stirring in an homogenizer (T18 digital ULTRA-TURRAX, IKA, Baden-Württemberg, Germany) with different mass ratios in the starch solution containing ammonium persulfate and N,N-methylenebisacrylamide. Then, the mixed solution was heated to 95 °C and kept for 2 h under a magnetic stirrer at 1000 rpm, and the gel could be bonded to form a stable gel. After cooling to room temperature, the gel was poured into a mold for liquid nitrogen freeze casting. Then, the frozen samples were sublimated for 3 days at −80 °C and 2 Pa pressure using a vacuum freeze dryer, and the ceramic fiber/starch aerogel was obtained. Finally, the obtained aerogel was heated in a tubular furnace at 600 °C for 1 h (argon atmosphere, heating rate of 1 °C min^−1^), and the carbonized ceramic fiber@amylopectin-derived carbon (CCF-ADC) aerogel was obtained. For comparison experiments, CCF-ADC samples with different mass ratios (1:0, 1:1, 1:1.5, 1:2) were prepared and named ADC, CCF-ADC1, CCF-ADC1.5, and CCF-ADC2, respectively.

### 2.4. Characterizations

The ceramic crystal structure was detected by an X-ray diffractometer (XRD, Miniflex 600 instrument, Rigaku, Tokyo, Japan) with Cu Kα radiation at a scanning speed of 2°min^−1^. The microstructure and element distribution state of the material were obtained by scanning electron microscopy (SEM, Apreo S LoVac, Thermo Fisher Scientific, Waltham, MA, USA) and an energy dispersive spectrometer (EDS). The ultraviolet/visible/near-infrared diffuse reflectance of the material was determined by an ultraviolet/visible/near-infrared spectrophotometer (UV-Vis-NIR spectrophotometer, Cary 5000, Agilent, Santa Clara, CA, USA). The appropriate amount of samples were filled in the powder pool and flattened. Using BaSO_4_ as the baseline standard, the light absorption properties of the samples were measured in the wavelength range of 200–2500 nm. The xenon lamp (PLS-SXE 300D, PerfectLight, Beijing, China) was used as an analog light source, and the infrared thermal imaging instrument (UTI160V, UNI-T, Dongguan, China) was used to perform infrared thermal imaging on the samples. The light intensity was detected by a light intensity meter (PL-MW2000, PerfectLight, Beijing, China). The density and porosity of aerogels were tested by the Archimedes drainage method (pure water (22 °C), NewClassic-ME0, METTLER TOLEDO, Columbus, OH, USA), and the specific surface area and pore size distribution of aerogels were tested by the BET method (ASAP 2020M + C, Micromeritics, Norcross, GA, USA).

### 2.5. Solar-Driven Interfacial Evaporation Test Process

**Figure 1:**
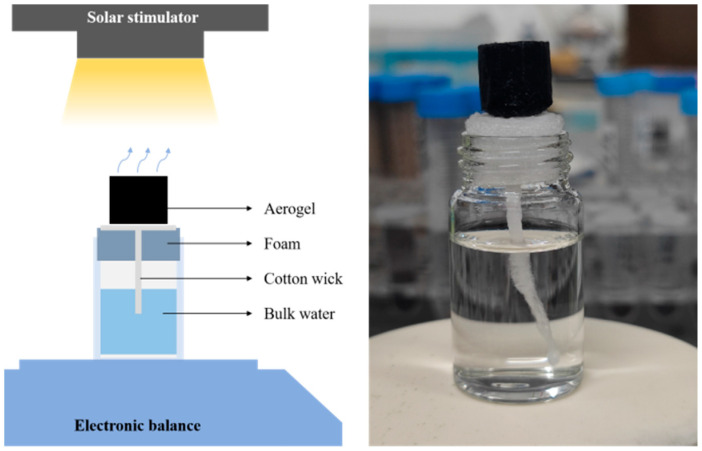
Schematic diagram and digital picture of solar evaporator.

Evaporation tests were conducted on a laboratory-built device (Figure 1). The sample was placed on a degreased cotton with the same bottom area as the evaporator, and the degreased cotton was placed in the polyethylene foam insulation layer to contact the water surface, thus forming a channel for the transmission of the solution. This design effectively isolates the conduction loss caused by the flow of water inside the material and the errors caused by the light energy radiation on the surface of other materials [40]. Using the PerfectLight PLS-SXE 300D xenon lamp as the simulated light source, the light intensity received by the material was controlled by adjusting the working current of the light source and the distance between the sample and the light source. The light intensity was detected using a PerfectLight PL-MW2000 photointensity meter, and the infrared temperature of the sample was measured using a UNI-T UTI160V infrared thermal imager to monitor the surface temperature change of the material under illumination and record the illumination time-temperature data. At a fixed light intensity, the change in the mass of the solution over time was obtained through the numerical change of the electronic balance to calculate the evaporation rate.

### 2.6. Calculation of Light Absorption Rate and Photothermal Conversion Efficiency

The solar energy absorption rate ($a_{s}$) of opaque materials can be calculated according to the following equation [41]:

(1) $$a_{s}=\int_{0.28\mu m}^{2.5\mu m}[1-R\left(\lambda \right)]P_{sun}\left(\lambda \right)d\lambda /\int_{0.28\mu m}^{2.5\mu m}P_{sun}\left(\lambda \right)d\lambda$$

$R\left(\lambda \right)$ is the measured value of sample reflectance, and $P_{sun}\left(\lambda \right)$ is the solar radiation (AM 1.5).

The evaporation rate can be calculated using the following equation [42]:

(2) $$m_{light}=\Delta m/A\times t$$

$m_{light}$ is the evaporation rate under light (kg m^−2^ h^−1^), A is the evaporation area (m^2^), ∆m is the change in water weight (kg), and t is the irradiation time (h).

The solar energy conversion efficiency $\left(\eta \right)$ is calculated according to the following equation [39,43]:

(3) $$\eta =\overset{•}{m}H_{LV}/C_{opt}q_{0}$$

$\dot{m}$ is the evaporation rate of the material under light ($\dot{m}=m_{light}-m_{dark}$, $m_{dark}$ = 0.2266 kg m^−2^ h^−1^ in our work), $H_{LV}$ is the equivalent evaporation enthalpy of water in the evaporator, which is the sum of the enthalpy of phase change (2257 kJ kg^−1^) and the sensible heat, where the specific heat capacity of water is 4.18 kJ kg^−1^ K^−1^, $C_{opt}$ is the optical concentration, and $q_{0}$ is the power density of light (1.0 sun = 1 kW m^−2^).

## 3. Results and Discussion

### 3.1. Preparation and Characterization of Ceramic Fiber@Biomass-Derived Carbon Composite Aerogels

**Figure 2:**
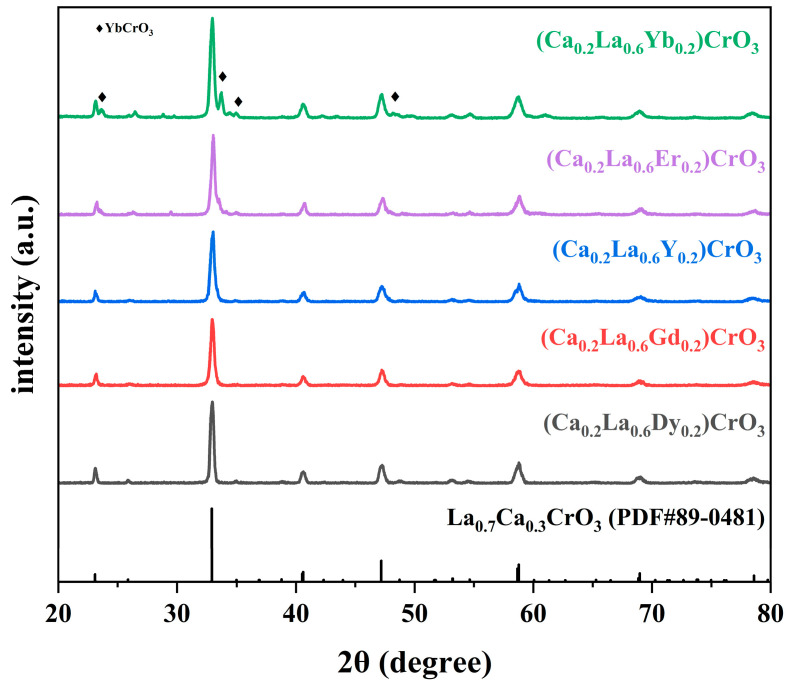
XRD images of (Ca_0_._2_La_0_._6_Yb_0_._2_)CrO_3_, (Ca_0_._2_La_0_._6_Er_0_._2_)CrO_3_, (Ca_0_._2_La_0_._6_Y_0_._2_)CrO_3_, (Ca_0_._2_La_0_._6_Gd_0_._2_)CrO_3_, (Ca_0_._2_La_0_._6_Dy_0_._2_)CrO_3_ ceramic fibers calcined at 1000 °C.

The elements Gd, Y, Dy, Er, and Yb, which are relatively abundant among heavy rare earth elements, were selected for doping with the A-bit light rare earth La via entropy regulation. These five rare earth elements have smaller ionic radii and chemical properties similar to those of La in crystals, which induced lattice distortion and increased the concentration of Cr^6+^ sites, thereby enhancing light absorption [44,45]. By introducing other rare earth elements with similar properties to replace La in the lattice through entropy regulation within the same family, the entropy of the material was increased along with the degree of system disorder, which ultimately increased light absorption [46]. As shown in Figure 2, (Ca_0_._2_La_0_._6_Yb_0_._2_)CrO_3_, (Ca_0_._2_La_0_._6_Er_0_._2_)CrO_3_, (Ca_0_._2_La_0_._6_Y_0_._2_)CrO_3_, (Ca_0_._2_La_0_._6_Gd_0_._2_)CrO_3_, and (Ca_0_._2_La_0_._6_Dy_0_._2_)CrO_3_ samples were synthesized and calcined at 1000 °C, then characterized with XRD. The results for all samples except (Ca_0_._2_La_0_._6_Yb_0_._2_)CrO_3_, which contained a small amount of YbCrO_3_ impurity, were consistent with the standard card (JCPDS #89-0481 [47]), demonstrating a single-phase cubic perovskite structure.

**Figure 3:**
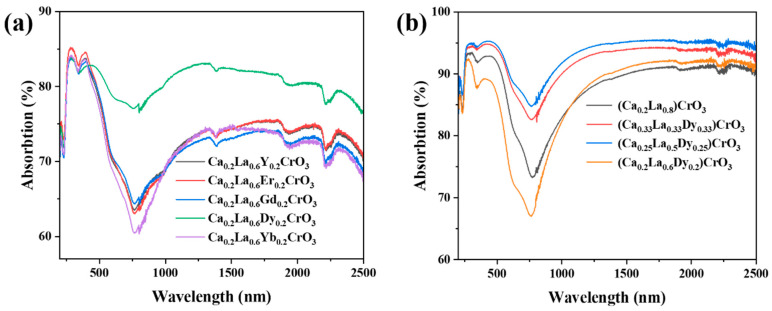
(**a**) Light absorption spectra of ceramics doped with Gd, Y, Dy, Er, and Yb in A-position, (**b**) light absorption effects of (Ca_0_._25_La_0_._5_Dy_0_._25_)CrO_3_, (Ca_0_._2_La_0_._6_Dy_0_._2_)CrO_3_, (Ca_0_._33_La_0_._33_Dy_0_._33_)CrO_3_, and (Ca_0_._2_La_0_._8_)CrO_3_ with different Dy doping amounts at 1000 °C.

Figure 3a shows that Gd-, Y-, Dy-, Er-, and Yb-doped (Ca_0_._2_La_0_._6_Re_0_._2_)CrO_3_ exhibited strong light absorption. The ceramic fibers doped with Gd, Y, Er, and Yb displayed similar absorption data at various wavelengths, indicating consistent optical properties for the substrates. Among them, (Ca_0_._2_La_0_._6_Dy_0_._2_)CrO_3_ exhibited the highest light absorption efficiency, suggesting that Dy doping increased the light absorbance of the lanthanum calcium chromate. Doping with Dy played pivotal roles [45,48,49,50,51]: (1) Doping with Dy increased the free carrier concentration in (Ca_x_La_1−x_)CrO_3_, which significantly increased the free carrier absorption coefficient; (2) introduction of the dopant atoms distorted the lattice structure, leading to reduced symmetry and increased dipole moments as well as disruption in the periodicities of the lattice vibrations, thereby strengthening the vibration absorption capacities of the materials; (3) the presence of charged impurities also contributed to the absorption of all lattice frequencies, resulting in higher surface absorption coefficients and optical absorbances; and (4) the impurities inhibited grain growth and decreased or increased the densities of the crystal particles, thus reducing the scattering coefficients and increasing the overall absorbances of the materials. Therefore, further investigations were conducted on samples doped with different amounts of Dy to explore their light absorbances, which are depicted in Figure 3b. The results indicated that (Ca_0_._25_La_0_._5_Dy_0_._25_)CrO_3_ absorbed more light than the other compositions tested herein. Therefore, (Ca_0_._25_La_0_._5_Dy_0_._25_)CrO_3_ ceramic fibers were selected as the photothermal conversion material for the solar interface evaporator in this study.

**Figure 4:**
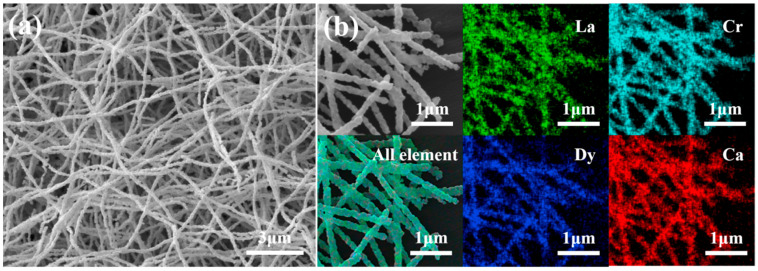
(**a**) SEM image and (**b**) EDS image of (Ca_0_._25_La_0_._5_Dy_0_._25_)CrO_3_ ceramic fiber.

The (Ca_0_._25_La_0_._5_Dy_0_._25_)CrO_3_ ceramic fibers were subjected to SEM and EDS studies to investigate the microstructures and elemental distributions of the synthesized fibers. Figure 4a shows SEM images revealing that the diameters of the ceramic fibers were approximately 100 nm, with each fiber consisting of a series of smaller grains. The surfaces of the fibers appeared rough yet predominantly continuous in nature. Figure 4b shows EDS images demonstrating homogeneous mixing of the components, which, when combined with XRD crystal structure data, confirmed doping of Dy into the calcium lanthanum chromate perovskite structure.

**Figure 5:**
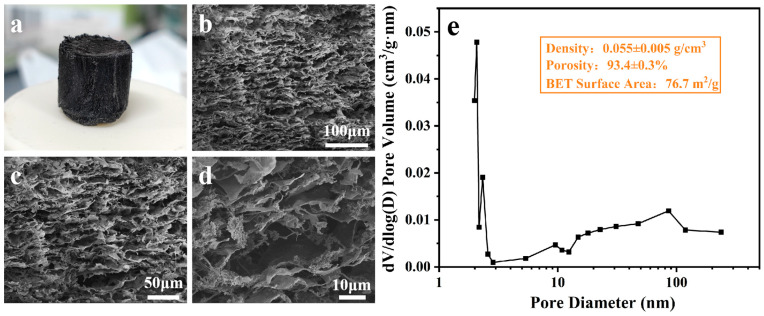
(**a**) Digital image of CCF-ADC2 sample, (**b**–**d**) SEM images of through-hole sections of CCF-ADC2 sample at different magnifications, (**e**) pore size distribution, density, porosity, and BET surface area.

Figure 5a shows a digital image of sample CCF-ADC2, and Figure 5b–d shows SEM images of sample CCF-ADC2 with different magnifications. As shown in the figures, the prepared ceramic fiber composite aerogel had a regularly arranged macroporous structure, and the fibers were effectively bonded to the carbonaceous inner walls. Through the BET test, the aerogel also has microporous and mesoporous structures (see Figure 5e). The density of the aerogel is 0.055 ± 0.005 g/cm^3^, and it has high porosity (93.4 ± 0.3%) and a regular pore structure, which could efficiently transport bottom water to the evaporation interface and prevent heat from being transferred downward into the water body at the same time. In addition, when sunlight penetrates the surface of the material, it will be reflected and refracted multiple times in the pores, greatly increasing the light absorption capacity and synergistically promoting the light-to-heat conversion performance of the ceramic fibers, thus increasing the overall light absorptivity of the composite aerogel [52].

**Figure 6:**
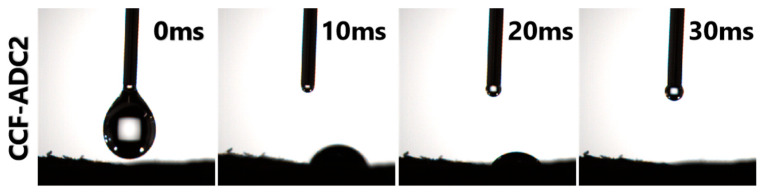
CCF-ADC2 water contact angle test.

The contact angle of sample CCF-ADC2 shown in Figure 6 demonstrated rapid penetration of water droplets into the material within a short time (30 ms), indicating hydrophilicity and efficient water transport of the composite aerogel. This ensured swift wetting of the ceramic fiber@carbon composite aerogel during interface evaporation, facilitating continuous transport from the bottom to the top surface for evaporation via capillary action. Moreover, the three-dimensional pore structure of the composite aerogel provided ample space for water vapor escape, while the high specific surface area offered abundant contact sites, thereby reducing the water evaporation enthalpy [39] and increasing interface evaporation.

### 3.2. Solar Evaporation Capabilities of Ceramic Fiber@Biomass-Derived Carbon Composite Aerogels

**Figure 7:**
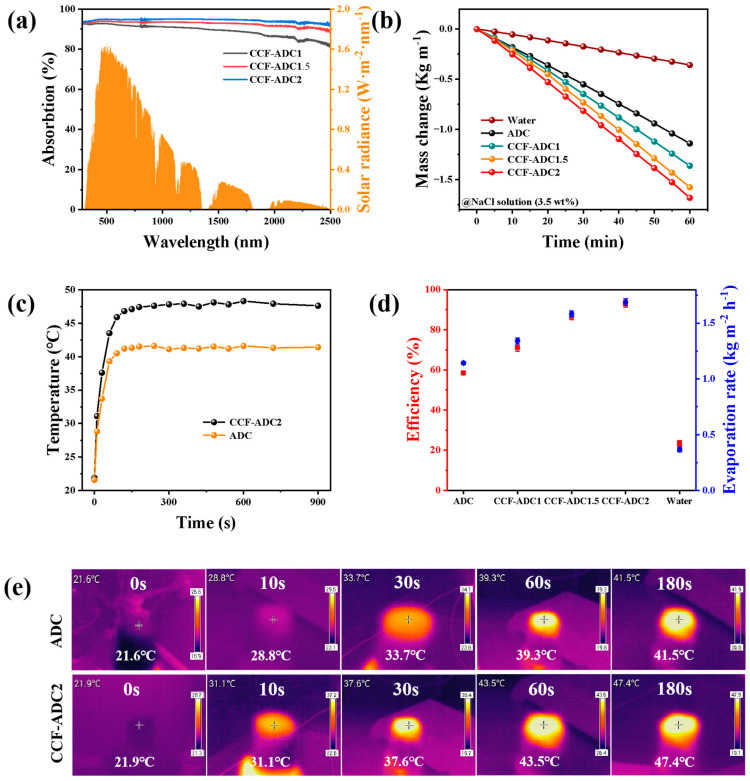
(**a**) Light absorption performance test and standard solar spectrum of CCF-ADC1, CCF-ADC1.5, and CCF-ADC2 samples, (**b**) the mass change of samples with different ceramic fiber contents in simulated seawater (3.5 wt% NaCl) within 60 min at 1.0 sun, (**c**) the surface temperature-time change curve of aerogels, (**d**) the evaporation rate and energy efficiency of each sample in simulated seawater at 1.0 sun, (**e**) the surface infrared image of aerogels during illumination.

An ideal photothermal conversion material should exhibit efficient solar energy absorption. Therefore, we studied solar absorption by the ceramic fiber-carbon composite aerogels. Surprisingly, as shown in Figure 7a, CCF-ADC1, CCF-ADC1.5, and CCF-ADC2 exhibited high light absorption efficiencies of 91.43%, 93.44%, and 94.72%, respectively, over a wide wavelength range (280–2500 nm). When sunlight illuminated the surface of a composite aerogel, the three-dimensional pore structure reflected the light multiple times, thereby increasing the amount of solar energy absorbed. Additionally, the composite aerogel also showed a rapid photothermal response. As shown in Figure 7c,e, the top temperature increased significantly within 30 s and reached the highest temperature within 2 min to provide temperature balance, and the highest temperature was 48.3 °C. Due to enhanced nonradiative relaxation of the ceramic fibers on the semiconductor surface, the temperature of the composite material was 6.7 °C higher than that of a biomass-derived carbon aerogel (41.6 °C). In addition, after the light source was turned off, the surface rapidly cooled to room temperature after several minutes, which again demonstrated the rapid photothermal response of the composite aerogel.

The water masses of the composite aerogels changed with varying ceramic fiber contents in 3.5 wt% simulated seawater, and the changes occurring within 60 min at an irradiation intensity of 1.0 sun are depicted in Figure 7b. The figure clearly illustrates the different water evaporation capacities among different samples synthesized under identical conditions, indicating that CCF-ADC2 exhibited more efficient water evaporation than the other samples. Notably, CCF-ADC2 demonstrated the highest evaporation rate of 1.68 kg m^−2^ h^−1^, which was 1.1 times, 1.25 times, 1.5 times, and 4.8 times greater than those of CCF-ADC1.5, CCF-ADC1, ADC, and water, respectively (Figure 7b,d). This outcome was consistent with the higher light absorptivity and greater evaporation efficiency observed for CCF-ADC2.

### 3.3. Evaporator Stability Test

**Figure 8:**
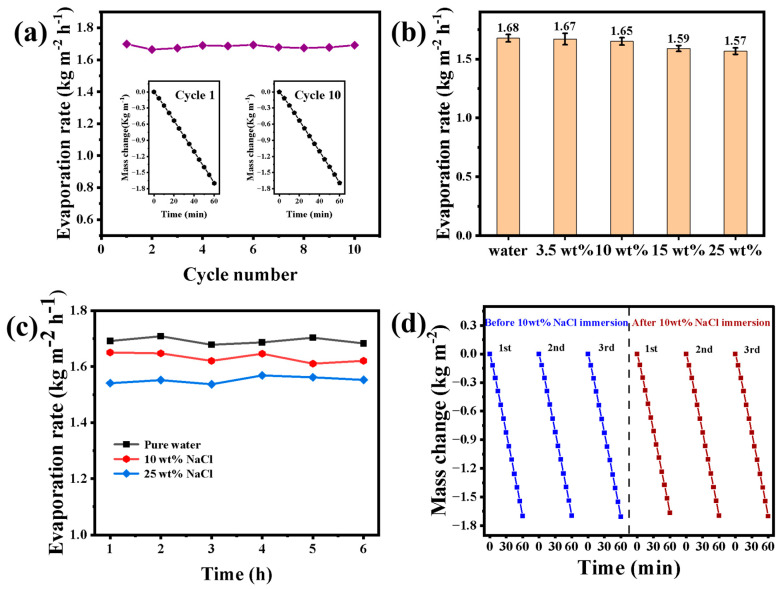
(**a**) Evaporation rates of CCF-ADC2 in 3.5 wt% simulated seawater for 10 cycles under 1.0 sun irradiation (inset: water mass changes during the first and tenth cycles), (**b**) evaporation rates of CCF-ADC2 in solutions with different salt concentrations, (**c**) evaporation rates of CCF-ADC2 in 10 wt% and 25 wt% NaCl solutions as a function of irradiation time under 1.0 sun irradiation, (**d**) simulated seawater evaporation effects of CCF-ADC2 after immersion in high concentration brine (10 wt% NaCl) for 10 days under 1.0 sun irradiation.

To verify the evaporation stability of CCF-ADC2, the cycling performance was determined at 1 kW m^−2^. Simulated seawater (3.5 wt%) was used as the evaporation medium in these experiments. The evaporation rate obtained by calculating the mass change was used to evaluate the performance of the absorber. A total of 10 cycles were conducted, each lasting 60 min, and the water was renewed after each cycle to reduce the impact of salinity changes on the experimental process. As shown in Figure 8a, in the solar-driven interfacial evaporation system, CCF-ADC2 maintained stable performance in the simulated seawater without obvious fluctuations after multiple cycles, and no obvious changes or salt precipitation occurred on the sample surface. Figure 8b shows that CCF-ADC2 exhibited relatively stable and high evaporation rates in solutions with different concentrations, which confirmed the reliability and consistency of the material in interfacial evaporation. Figure 8c shows the hourly evaporation rates for CCF-ADC2 during continuous evaporation of pure water and in 10 wt% and 25 wt% salt solutions. Although the material showed different evaporation rates for different concentrations, it maintained stable and reliable evaporation under the same conditions. Additionally, we also tested the evaporation rates for CCF-ADC2 in concentrated brine (10 wt% NaCl) for 10 days before and after soaking, and no significant changes were observed, confirming that the material was stable (Figure 8d).

**Figure 9:**
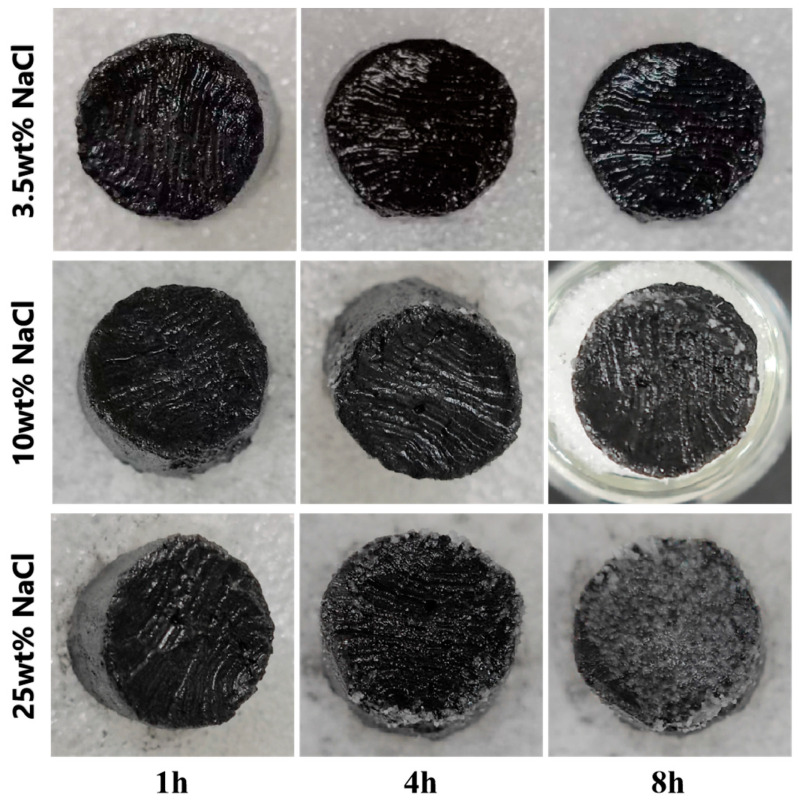
Changes in surface salt crystallization of CCF-ADC2 in 25 wt% NaCl solution over time under 1.0 solar intensity irradiation.

Figure 9 clearly shows that CCF-ADC2 continued to provide evaporation without obvious salt crystallization from 3.5 wt% simulated seawater, indicating that the material showed excellent long-term performance with low concentrations of brine. When the solution concentration reached 10 wt%, a few grains began to precipitate after a long period of evaporation; even in ultrahigh concentrations of brine (25 wt% NaCl), the material still maintained a high evaporation rate for a short time without obvious crystallization. This indicated that CCF-ADC2 has good salt resistance, which was attributed to the fact that in the evaporation process, the salt redissolved in the solution and was transferred to the bottom water through the pores; this ensured that salt crystallized on the surface did not affect the light absorption efficiency and continuously kept the surface clean [52].

**Figure 10:**
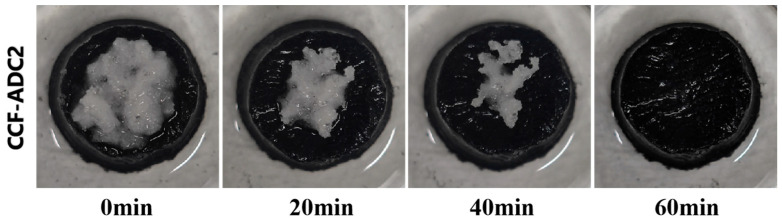
Re-dissolution of solid NaCl crystallization on the surface of CCF-ADC2.

When continuous evaporation of an ultrahigh-concentration salt solution exceeded the salt transport rate of the material, large blocks of salt were inevitably deposited on the surface. Figure 10 clearly shows that when the bottom of the material was immersed in the solution, CCF-ADC2 dissolved the surface salt crystals and transported them to the solution through the internal pores; it completely removed the surface crystals in a short time, thus providing excellent resistance to salt precipitation and enabling self-cleaning. In daily use, evaporators operate efficiently under sufficient sunlight during the day, and the surface salt crystals will naturally dissolve into the solution at night. This natural cycle of crystallization during the day and dissolution at night enables long-term, stable evaporation without manual maintenance.

## 4. Conclusions

In this study, we successfully prepared a (Ca_0_._25_La_0_._5_Dy_0_._25_)CrO_3_ ceramic fiber@biomass-derived carbon aerogel with excellent stability, hydrophilicity, high light absorption performance, and through-hole structure via electrospinning and freeze-drying technology. The aerogel was employed as an evaporator in simulated seawater (3.5 wt%) under 1 kW m^−2^ light intensity, exhibiting the highest light absorption efficiency of 94.72% and evaporation rate of 1.68 kg m^−2^ h^−1^. By analyzing infrared images and absorption spectroscopy data, we found that the content of (Ca_0_._25_La_0_._5_Dy_0_._25_)CrO_3_ ceramic fiber played a crucial role in determining both solar absorption rate and evaporation rate. The CCF-ADC2 sample, with a ceramic fiber to amylopectin mass ratio of 2:1, exhibited a stable maximum temperature of 48.3 °C under an irradiation intensity of 1 kW m^−2^. Moreover, it maintained a steady evaporation rate of 1.57 kg m^−2^ h^−1^ in high-concentration brine (25 wt%) under the same light intensity and demonstrated excellent resistance to salt crystallization. In stability tests, CCF-ADC2 showed remarkable durability, even after multiple repeated recycling cycles. Overall, the novel (Ca_0_._25_La_0_._5_Dy_0_._25_)CrO_3_ ceramic fiber@biomass-derived carbon aerogel developed in this study holds significant potential for application as a solar-driven interfacial evaporator in seawater desalination and sewage treatment.

## Data Availability

Data are contained within the article.
